# Living Donor Liver Transplantation for Hepatocellular Carcinoma: It Is All about Donors?

**Published:** 2013-11-01

**Authors:** R. F. Saidi, Y. Li, S. A. Shah, N. Jabbour

**Affiliations:** 1 *Division of Organ Transplantation, Department of Surgery, Alpert Medical School of Brown University, Providence, USA*; 2 *Division of Organ Transplantation, Department of Surgery, University of Massachusetts Medical School, Worcester, MA, USA*

**Keywords:** Living donor, Liver transplantation, Hepatocellular carcinoma, Outcome assessment (health care)

## Abstract

Background: Live-donor liver transplantation (LDLT) is a valuable option for patients with hepatocellular carcinoma (HCC) as compared with deceased-donor liver transplantation (DDLT); the tumor could be eradicated early.

Methods: Herein, we reviewed the outcome of adult patients with HCC who underwent LDLT from 1990 to 2009 in the USA, as reported to United Network for Organ Sharing.

Results: Compared to DDLT (n=5858), patients who underwent LDLT for HCC (n=170) were more likely to be female (43.8% *vs* 23.8%), younger (mean age 48.6 *vs* 54.9 years) and have more tumors outside Milan criteria (30.7% *vs* 13.6%). However, the recipients of LDLT for HCC had a significantly shorter mean wait time before transplantation (173 *vs* 219 days; p=0.04). The overall allograft and patient survival were not different, though more patients in LDLT group were outside Milan criteria. Since implementation of the MELD exception for HCC, DDLT for HCC has increased form 337 (2.3%) cases in 2002 to 1142 (18.7%) in 2009 (p<0.001). However, LDLT for HCC has remained stable from 16 (5.7%) in 2002 to 14 (9.2%) in 2009 (p=0.1). Regions 1, 5 and 9 had the highest rate of LDLT for HCC compared to other regions.

Conclusions: LDLT can achieve the same long-term outcomes compared to DDLT in patients with HCC. The current MELD prioritization for HCC reduces the necessity of LDLT for HCC except in areas with severe organ shortage.

## INTRODUCTION

Hepatocellular carcinoma (HCC) ranks third among the leading causes of cancer death. It is the fifth most prevalent cancer worldwide [1]. Most cases of HCC (80%–90%) arise against a background of cirrhosis [2]. Liver transplantation (LT) offers the best chance of curing HCC that is confined to the liver because it provides a complete oncological resection, removes the diseased liver for further cancer development, and corrects the underlying liver dysfunction [3-5]. LDLT for HCC offers several advantages: shorter wait times and the possibility of optimizing the pre-transplantation bridge treatment of the tumor.

Early reports revealed higher rates of cancer recurrence and inferior patient survival after LDLT *vs* deceased donor liver transplantation (DDLT) [6-9]. The rate of recurrence after LDLT may be higher because the growth factors that mediate the regeneration of the hemiliver after LDLT may potentiate HCC recurrence or an aggressive or rapidly progressive HCC biology may not be recognized during the decreased wait time for LDLT [10-12]. The present study was therefore, undertaken to investigate the outcomes of LDLT and DDLT for HCC as reported to United Network for Organ Sharing (UNOS/OPTN). 

## MATERIALS AND METHODS

The data was collected from the UNOS/OPTN as reported between 1990 and 2009. Only adult patients who underwent liver transplantation (LDLT or DDLT) for HCC were included in the study. We also examined the use of LT for HCC in different UNOS regions.

We collected the following information under recipient in both groups: patient age, sex, MELD score, hospital length of stay, wait time prior to transplantation, tumor status (within *vs* outside Milan criteria) allograft and patients survival. Donor age and sex were collected on the donors. Missing values were imputed with the mean values.

χ^2^ and *Student’s t* tests were used for comparison of proportions and means, respectively. Allograft and patient survival was the primary outcome measured. Kaplan-Meier survival analysis was used for allograft and patient survival estimates. Variables with more that 20% missing values were excluded from data analysis. 

This study was reviewed by the University of Massachusetts Medical School Institutional Review Board (IRB) and deemed appropriate for exemption from IRB oversight as no personal identifiers were used among datasets.

## RESULTS

From 1990 to 2009, 6028 patients underwent LT for HCC—5858 (97.1%) had DDLT and 170 (2.9%) LDLT. Donors and recipients characteristics are shown in Table 1. Compared to patients who underwent DDLT for HCC, patients who underwent LDLT were more likely to be female (43.8% *vs* 23.8%), younger (mean age: 48.6 *vs* 54.9 years) and have tumor outside Milan criteria (30.7% *vs* 13.6%). However, the recipients of LDLT for HCC had a significantly shorter mean wait time before transplantation (173 *vs* 219 days).

**Table 1:** Donors and recipients characteristics

Variables	HCC-LDLT n=170	HCC-DDLT n=5858	p
Recipient female (%)	75 (43.8%)	1394 (23.8%)	0.004
Recipient age	48.6+-17.4	54.9±11.3	0.009
Donor female (%)	77 (45.8%)	2272 (38.8%)	0.3
Donor age	37.7+-9.2	40.9±18.1	0.004
MELD	11.1+-5.2	14±7.5	0.1
Length of stay	14.6+-11.8	14.9±17.4	0.6
Waiting time	173.2+-274.3	219.9±426.5	0.04
Tumor Status			
Milan	104 (61.5%)	4557 (77.8%)	<0.001
Outside Milan	52 (30.7%)	796 (13.6%)	
Unknown	14 (7.8%)	505 (8.6%)	

**Figure 1:**
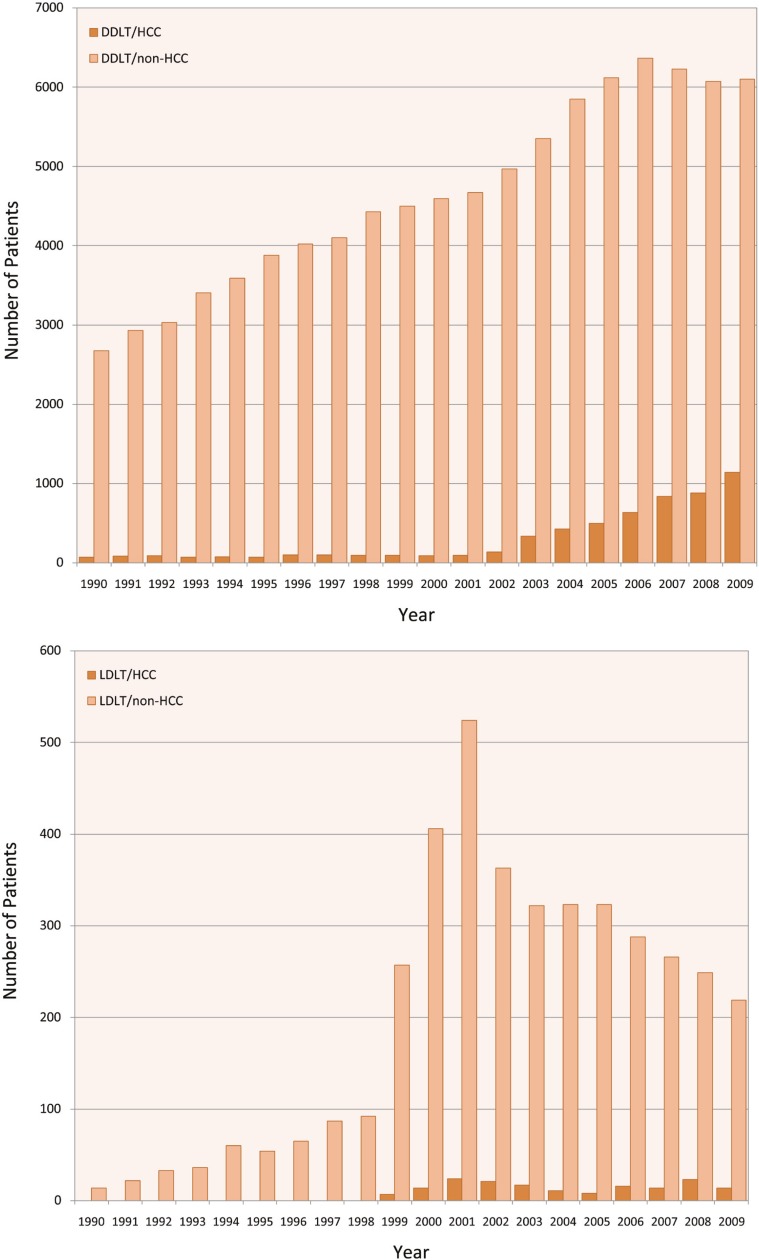
Number of patients underwent Top) DDLT, and Down) LDLT comparing HCC vs non-HCC from 1990–2009

**Figure 2:**
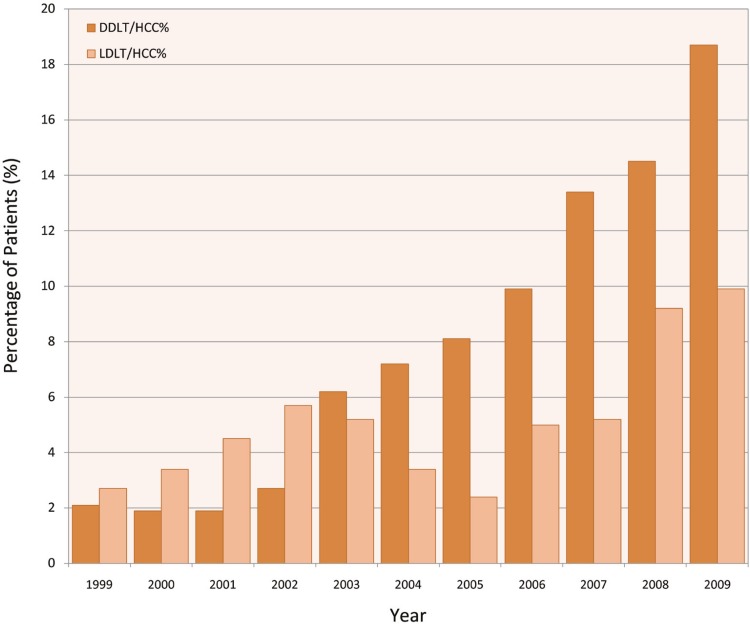
Relative frequency of LDLT and DDLT which was dedicated to HCC patients

**Figure 3:**
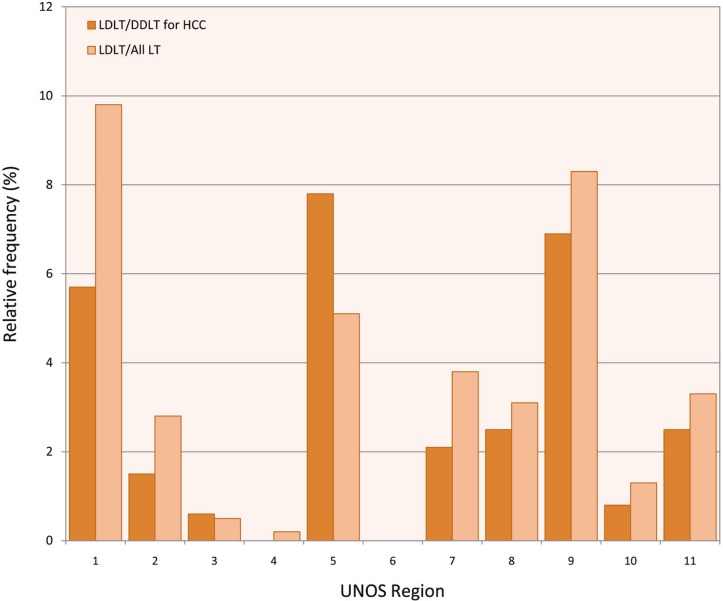
Frequency of LDLT vs DDLT for HCC in 11 UNOS regions

**Figure 4:**
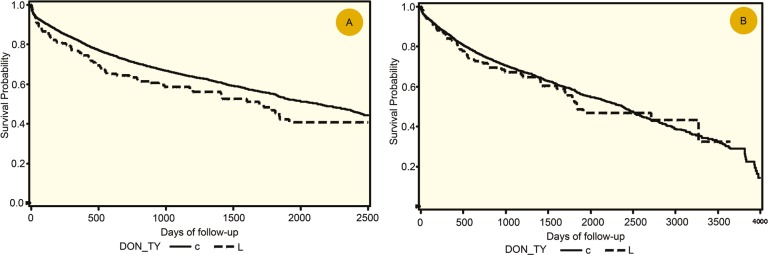
A) Allograft and B) patient survival compairing DDLT (solid line) and LDLDT (dashed line) for HCC

We noted an increase in the number of DDLT for HCC since implementation of MELD and HCC exception in 2002. From 1990–2001, the number of DDLT for HCC remained stable (Fig 1). However, this number has been on the rise since 2002. Since implementation of MELD exception for HCC, DDLT for HCC has increased from 337 (2.3%) cases in 2002 to 1142 (18.7%) in 2009 (p<0.001). However, LDLT for HCC (Figs 2 and 3) has remained stable from 16 (5.7%) in 2002 to 14 (9.2%) in 2009 (p=0.1). We noted a difference in utilization of LDLT for HCC patients in 11 UNOS regions. Regions 1, 5 and 9 had the highest rate of LDLT for HCC compared to other regions (Fig 4).

**Figure 5:**
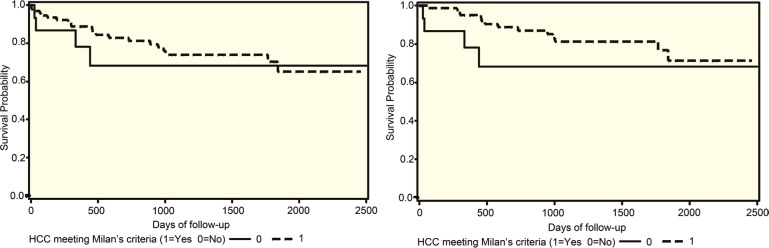
Left) Allograft and Right) patients survival for patients who underwent LDLT for HCC comparing tumor within (dashed line) and outside (solid line) Milan criteria

The overall allograft and patients’ survival were comparable in both groups despite the fact that one-third of patients in LDLT group were outside Milan criteria (Fig 5). There was no difference in survival comparing tumor with *vs* outside Milan criteria (Fig 5).

## DISCUSSION

Milan criteria was accepted by UNOS as a basis for organ allocation for patients with HCC—patients meeting the criteria can have one tumor <5 cm in diameter or up to three HCCs, the biggest not exceeding 3 cm. Therefore, additional MELD points were awarded to patients with HCC according to tumor size and number as of February 2002 [13]. Since then, the number of DDLT for HCC is on the rise. On the other hand, the number of LDLT for HCC has remained relatively stable. Due to a current allocation system for DDLT for HCC patients, it is not surprising to see that one-third of patients in LDLT group had a tumor outside Milan criteria.

This study compared the results of LDLT and DDLT for patients with HCC. In contrast to some previous reports [5-9], we found no evidence of a poor outcome in patients after LDLT *vs* DDLT. Toronto group reported HCC recurrence rates at 1, 3, and 5 years, of 8.8%, 10.7%, and 15.4%, respectively, for the LDLT group, and 7.5%, 14.8%, and 17.0%, for the DDLT recipients [14]. Adult-to-Adult Living Donor Liver Transplantation (A2ALL) cohort study in the USA showed that the 3-year HCC recurrence rate was 29% for LDLT and 0% for DDLT [8]. Similarly, Lo, *et al*, reported a higher rate of recurrence at five years in LDLT group (29%) *vs* DDLT group (0%) [7]. In a more recent study, Vakili, *et al*, found that LDLT recipients experienced HCC recurrence more often than DDLT recipients (29% *vs* 12%) [9]. We found that despite the fact that one-third of HCC patients in the LDLT group had tumors outside Milan criteria, the survival rate was comparable to that in DDLT group. There was also no difference in LDLT for HCC comparing patients within or outside Milan criteria. This again raises the question that whether or not Milan criteria is restrictive and can be safely expanded [15].

The inferior outcomes with LDLT *vs* DDLT for HCC that were identified in the latter comparative cohort studies and in other case series may be attributed to differences in the population case mixes [5-9]. For instance, in the A2ALL study, 15% of the patients in the LDLT group had poorly differentiated tumors [8]. The study by Lo, *et al*, likewise had a higher proportion of LDLT patients with poorly differentiated tumors (19%) [7]. Another poor prognostic indicator was prevalent in the patients studied by Vakili, *et al*—46% of the tumors in the LDLT group had microvascular invasion [9].

We also noted a significant difference in different UNOS regions in utilization of LDLT for HCC. Some of the observed differences in the number of LDLTs may also be explained by regional variations in deceased donor organ availability and the subsequent use of living donors for HCC patients [16]. Regions 1, 5 and 9 had the highest rates of LDLT/DDLT for HCC. A recent study showed that these three regions had lower rates of LT within three months for patients with MELD>20 [17]. For example, the percentage of patients who get transplanted with MELD >20 in regions 1, 5 and 9 were 9.6%, 11.8%, and 16.4%, respectively, which were lowest compared to other regions [17].

Since the implementation of the MELD allocation system in 2002, there has been a marked improvement in the equitable allocation of liver allografts with respect to race and ethnicity; however, disparities based on sex and geography appear to remain [16]. The disparity in liver allocation and thus, the disparity in wait list mortality between males and females after the implementation of the MELD allocation system, have been attributed to multiple factors including the underestimation of the degree of renal dysfunction in female patients and the smaller size of female recipients, which is a disadvantage at the time of donor/recipient organ matching. In our study, there were more females in LDLT group compared to DDLT (43.8% *vs* 23.8%). A recent study showed that the use of living liver donation appears to be targeted at those patients who are disadvantaged for undergoing DDLT and/or reside in regions where a high MELD score is required to undergo DDLT [18]. Our study also showed that the UNOS regions with higher degree of competition and organ shortage had the highest rate of LDLT for HCC. 

This study has several limitations. First, it is a retrospective analysis of UNOS data. We recognize both potential advantages and limitations of this study that uses a large national database. However, the large sample size provides sufficient power to detect independent risk factors that may be missed by single-center studies. As with any analysis utilizing the UNOS database, our conclusions rely on the assumption that there is no systematic bias generated by reporting error or missing data. However, the primary endpoint for this analysis was allograft and patient survival, which is reliably captured in the UNOS database. Residual or unmeasured confounders that could impact allograft and patient survival include surgeon technique, differences in immunosuppression protocols, the fat content/quality of the allograft and center-specific practices. Second, detail data on the tumor characteristics, total number of lesions, size, pre-transplant HCC treatment, lymphovascular invasion and the recurrence rate were not available in the database. Third, we were not able to analyzed center-specific outcomes.

In conclusion, LDLT and DDLT provide similar outcomes for patients with HCC. LDLT can expand the donor pool, especially in areas with severe organ shortage.

## Notes

**FINANCIAL SUPPORT:** None.
